# Supplementary material for: Resolving the full spectrum of human genome variation using Linked-Reads
Source: Genome Res. 2019 Apr;29(4):635–45. doi: 10.1101/gr.234443.118 (PMC6442396; doi:10.1101/gr.234443.118)
Supplement: Supplemental Material [file supp_gr.234443.118_Supplemental_longranger-2.2.zip › longranger-2.2.tar.bz2!/lr2.2-public/lib/stan/cmdstan/stan/doc/api/html/agrad_2rev_2matrix_2log__softmax_8hpp_source.html]

Stan: src/stan/agrad/rev/matrix/log\_softmax.hpp Source File


|  |  |
| --- | --- |
|  | Stan  2.5.0  probability, sampling & optimization |


- Main Page
- Related Pages
- Namespaces
- Classes
- Files

- File List
- File Members

All Classes Namespaces Files Functions Variables Typedefs Enumerations Enumerator Friends Macros Pages

- src
- stan
- agrad
- rev
- matrix

log\_softmax.hpp

Go to the documentation of this file.

1 #ifndef STAN\_\_AGRAD\_\_REV\_\_MATRIX\_\_LOG\_SOFTMAX\_HPP

2 #define STAN\_\_AGRAD\_\_REV\_\_MATRIX\_\_LOG\_SOFTMAX\_HPP

3

4 #include <cmath>

5 #include <vector>

6 #include <stdexcept>

7 #include <stan/math/matrix/Eigen.hpp>

8 #include <stan/math/matrix/log\_softmax.hpp>

9 #include <stan/math/matrix/softmax.hpp>

10 #include <stan/agrad/rev/var.hpp>

11 #include <stan/math/error\_handling/matrix/check\_nonzero\_size.hpp>

12

13 namespace stan {

14  namespace agrad {

15

16  namespace {

17

18  class log\_softmax\_elt\_vari : public vari {

19  private:

20  vari\*\* alpha\_;

21  const double\* softmax\_alpha\_;

22  const int size\_; // array sizes

23  const int idx\_; // in in softmax output

24  public:

25  log\_softmax\_elt\_vari(double val,

26  vari\*\* alpha,

27  const double\* softmax\_alpha,

28  int size,

29  int idx)

30  : vari(val),

31  alpha\_(alpha),

32  softmax\_alpha\_(softmax\_alpha),

33  size\_(size),

34  idx\_(idx) {

35  }

36  void chain() {

37  for (int m = 0; m < size\_; ++m) {

38  if (m == idx\_)

39  alpha\_[m]->adj\_ += adj\_ \* (1 - softmax\_alpha\_[m]);

40  else

41  alpha\_[m]->adj\_ -= adj\_ \* softmax\_alpha\_[m];

42  }

43  }

44  };

45

46  }

47

48

59  inline Eigen::Matrix<var,Eigen::Dynamic,1>

60  log\_softmax(const Eigen::Matrix<var,Eigen::Dynamic,1>& alpha) {

61  using Eigen::Matrix;

62  using Eigen::Dynamic;

63

64  stan::math::check\_nonzero\_size("log\_softmax(%1%)",alpha,"alpha",(double\*)0);

65

66  if (alpha.size() == 0)

67  throw std::domain\_error("arg vector to log\_softmax() must have size > 0");

68  if (alpha.size() == 0)

69  throw std::domain\_error("arg vector to log\_softmax() must have size > 0");

70

71  if (alpha.size() == 0)

72  throw std::domain\_error("arg vector to log\_softmax() must have size > 0");

73

74  vari\*\* alpha\_vi\_array

75  = (vari\*\*) agrad::chainable::operator new(sizeof(vari\*) \* alpha.size());

76  for (int i = 0; i < alpha.size(); ++i)

77  alpha\_vi\_array[i] = alpha(i).vi\_;

78

79

80  Matrix<double,Dynamic,1> alpha\_d(alpha.size());

81  for (int i = 0; i < alpha\_d.size(); ++i)

82  alpha\_d(i) = alpha(i).val();

83

84  // fold logic of math::softmax() and math::log\_softmax() to save computations

85

86  Matrix<double,Dynamic,1> softmax\_alpha\_d(alpha\_d.size());

87  Matrix<double,Dynamic,1> log\_softmax\_alpha\_d(alpha\_d.size());

88

89  double max\_v = alpha\_d.maxCoeff();

90

91  double sum = 0.0;

92  for (int i = 0; i < alpha\_d.size(); ++i) {

93  softmax\_alpha\_d(i) = std::exp(alpha\_d(i) - max\_v);

94  sum += softmax\_alpha\_d(i);

95  }

96

97  for (int i = 0; i < alpha\_d.size(); ++i)

98  softmax\_alpha\_d(i) /= sum;

99  double log\_sum = std::log(sum);

100

101  for (int i = 0; i < alpha\_d.size(); ++i)

102  log\_softmax\_alpha\_d(i) = (alpha\_d(i) - max\_v) - log\_sum;

103

104  // end fold

105

106  double\* softmax\_alpha\_d\_array

107  = (double\*) agrad::chainable::operator new(sizeof(double) \* alpha\_d.size());

108

109  for (int i = 0; i < alpha\_d.size(); ++i)

110  softmax\_alpha\_d\_array[i] = softmax\_alpha\_d(i);

111

112  Matrix<var,Dynamic,1> log\_softmax\_alpha(alpha.size());

113  for (int k = 0; k < log\_softmax\_alpha.size(); ++k)

114  log\_softmax\_alpha(k) = var(new log\_softmax\_elt\_vari(log\_softmax\_alpha\_d[k],

115  alpha\_vi\_array,

116  softmax\_alpha\_d\_array,

117  alpha.size(),

118  k));

119  return log\_softmax\_alpha;

120  }

121

122

123  }

124 }

125

126 #endif

idx\_

const int idx\_

**Definition:** log\_softmax.hpp:23

stan::agrad::log\_softmax

Eigen::Matrix< fvar< T >, Eigen::Dynamic, 1 > log\_softmax(const Eigen::Matrix< fvar< T >, Eigen::Dynamic, 1 > &alpha)

**Definition:** log\_softmax.hpp:16

stan::agrad::sum

fvar< T > sum(const Eigen::Matrix< fvar< T >, R, C > &m)

**Definition:** sum.hpp:14

stan::agrad::vari

The variable implementation base class.

**Definition:** vari.hpp:28

var.hpp

softmax.hpp

alpha\_

vari \*\* alpha\_

**Definition:** log\_softmax.hpp:20

Eigen.hpp

log\_softmax.hpp

size\_

const int size\_

**Definition:** log\_softmax.hpp:22

stan::agrad::var

Independent (input) and dependent (output) variables for gradients.

**Definition:** var.hpp:27

stan::math::size

int size(const std::vector< T > &x)

**Definition:** size.hpp:11

softmax\_alpha\_

const double \* softmax\_alpha\_

**Definition:** log\_softmax.hpp:21

check\_nonzero\_size.hpp

stan::agrad::log

fvar< T > log(const fvar< T > &x)

**Definition:** log.hpp:15

stan::agrad::exp

fvar< T > exp(const fvar< T > &x)

**Definition:** exp.hpp:16

stan::math::check\_nonzero\_size

bool check\_nonzero\_size(const char \*function, const T\_y &y, const char \*name, T\_result \*result)

Return true if the specified matrix/vector is of non-zero size.

**Definition:** check\_nonzero\_size.hpp:33


---

[ Stan Home Page ]

*© 2011–2014,
Stan Development Team.*
